# Targeting mitophagy in Parkinson's disease

**DOI:** 10.1074/jbc.REV120.014294

**Published:** 2020-12-24

**Authors:** Emily H. Clark, Aurelio Vázquez de la Torre, Tamaki Hoshikawa, Thomas Briston

**Affiliations:** Hatfield Research Laboratories, Neurology Innovation Centre, Eisai Ltd, Hatfield, United Kingdom

**Keywords:** biomarker, Parkinson’s disease, mitochondria, mitophagy, drug discovery, PINK1, Parkin, ADP, adenosine diphosphate, AMP, adenosine monophosphate, ATP, adenosine triphosphate, cGMP, cyclic guanosine monophosphate, DA, dopaminergic, DAT, dopamine transporter, DUBs, deubiquitinases, ETC, electron transport chain, GBA, glucocerebrosidase, GFP, green fluorescent protein, HTS, high-throughput screen, IMM, inner mitochondrial membrane, IMS, intermembrane space, KR, kinetin riboside, MDVs, mitochondrially derived vesicles, MPP, mitochondrial processing peptidase, MTS, mitochondrial targeting sequence, NAD, nicotinamide adenine dinucleotide, PBMCs, peripheral blood mononuclear cells, PD, Parkinson’s disease, PET, positron emission tomography, PTMs, posttranslational modifications, SPECT, single-photon emission computed tomography, SNc, substantia nigra pars compacta, UA, Urolithin A

## Abstract

The genetics and pathophysiology of Parkinson’s disease (PD) strongly implicate mitochondria in disease aetiology. Elegant studies over the last two decades have elucidated complex molecular signaling governing the identification and removal of dysfunctional mitochondria from the cell, a process of mitochondrial quality control known as mitophagy. Mitochondrial deficits and specifically reduced mitophagy are evident in both sporadic and familial PD. Mendelian genetics attributes loss-of-function mutations in key mitophagy regulators *PINK1* and *Parkin* to early-onset PD. Pharmacologically enhancing mitophagy and accelerating the removal of damaged mitochondria are of interest for developing a disease-modifying PD therapeutic. However, despite significant understanding of both PINK1-Parkin-dependent and -independent mitochondrial quality control pathways, the therapeutic potential of targeting mitophagy remains to be fully explored. Here, we provide a summary of the genetic evidence supporting the role for mitophagy failure as a pathogenic mechanism in PD. We assess the tractability of mitophagy pathways and prospects for drug discovery and consider intervention points for mitophagy enhancement. We explore the numerous hit molecules beginning to emerge from high-content/high-throughput screening as well as the biochemical and phenotypic assays that enabled these screens. The chemical and biological properties of these reference compounds suggest many could be used to interrogate and perturb mitochondrial biology to validate promising drug targets. Finally, we address key considerations and challenges in achieving preclinical proof-of-concept, including *in vivo* mitophagy reporter methodologies and disease models, as well as patient stratification and biomarker development for mitochondrial forms of the disease.

## Mitochondria and Parkinson’s disease

Parkinson’s disease (PD) is a late-onset neurodegenerative disorder characterized by progressive loss of dopaminergic (DA) neurons of the substantia nigra pars compacta (SNc). Reduced dopaminergic innervation to the striatum leads to cardinal motor phenotypes, including resting tremor, bradykinesia, muscle rigidity, and postural instability. DA neurons have significant bioenergetic and metabolic requirements, with the consequence of acute vulnerability to mitochondrial stress (1). Indeed, mitochondrial dysfunction is a prominent pathological hallmark of both sporadic and familial PD (2, 3, 4, 5, 6, 7, 8). Mitochondria are multifunctional organelles contributing to a diverse range of cellular processes, including generation of adenosine triphosphate (ATP) *via* oxidative phosphorylation, lipid, and heme biosynthesis, Ca^2+^ signaling, and programmed cell death. Mitochondria are also highly dynamic, undergoing continuous cycles of fission and fusion, rapidly undergoing quality control checks, and adapting to the cellular environment. Damaged mitochondria are segregated from the healthy mitochondrial reticulum and eliminated through mitophagy, a complex pathway regulated by a series of posttranslational modifications (PTMs), culminating in recruitment of the autophagic machinery to dysfunctional mitochondria or mitochondrial fragments and their degradation *via* lysosomes (9). Mitochondrial failure and reduced mitophagy have been proposed as important components in determining pathological heterogeneity and selective vulnerability of specific brain regions in PD (6, 8).

**Figure 1:**
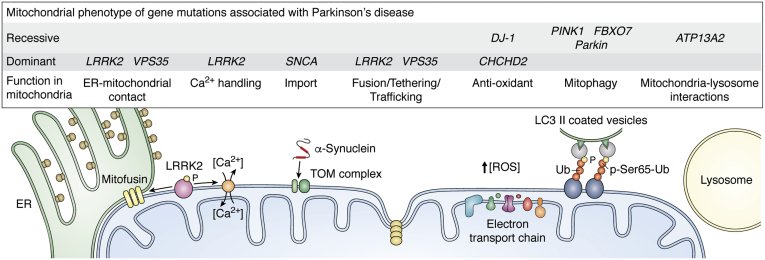
**Human genetics link mitochondria to PD.** Multiple genes associated with PD play a role in aspects of mitochondrial homeostasis. *LRRK2* mutations affect ER-Mitochondria tethering and Ca^2+^ homeostasis. α-synuclein interacts with the TOM complex, affecting mitochondrial import. Mutations in *VPS35* increase mitochondrial fragmentation, while mutations in *DJ-1* or *CHCHD2* are associated with increased ROS production. *PINK1*, *FBXO7*, and *Parkin* mutations cause defective mitophagy and *ATP13A2* mutations alter lysosomal function.

Monogenic PD strongly implicates mitochondria as central to disease pathogenesis (Fig. 1). Mutations in phosphatase and tensin homolog (PTEN)-induced kinase 1 (PINK1; encoded by *PARK6*) and Parkin (encoded by *PARK2*), two key mitophagy proteins, cause autosomal recessive early onset PD (EOPD) (3, 10, 11). Indeed loss-of-function mutations in *PINK1* and *Parkin* are the most common cause of PD in those under the age of 45 years, contributing to approximately 13% of cases (12). F-box only protein 7 (FBXO7; *PARK15*) is associated with autosomal recessive EOPD. FBXO7, serving as an adapter protein functioning within an E3-ubiqutin ligase complex to mediate both degradative and nondegradative protein ubiquitination, has multifunctional actions on mitochondria and can influence mitophagy through interactions with PINK1 and Parkin (13, 14, 15, 16). Finally, vacuolar protein sorting 13C (VPS13C; *PARK23*), mutation in which has also been associated with autosomal recessive EOPD, is partly localized to the outer mitochondrial membrane (OMM). Exactly how VPS13C mutation causes PD is uncertain. Loss of VPS13C, however, is associated with reduced mitochondrial membrane potential, effects on metabolism and altered mitochondrial morphology. Additionally, VPS13C has been proposed to function with PINK1 and Parkin to regulate mitochondrial clearance (17, 18).

Several genes are associated with autosomal dominant PD, including, coiled-coil-helix-coiled-coil-helix domain containing 2 (CHCHD2; *PARK22*) and vacuolar protein sorting protein 35 (VPS35; *PARK17*). CHCHD2 is a mitochondrial intermembrane space (IMS) protein, proposed to maintain mitochondrial cristae and integrity of electron transport chain (ETC) (19, 20). VPS35 is a retromer sorting protein, which regulates mitochondrial dynamics *via* modulation of mitofusin-2 (MFN-2) and mitochondrial ubiquitin ligase 1 (MUL1; also known as MAPL or MULAN) stability (21, 22, 23). Other major genes associated with autosomal dominant familial PD, leucine-rich repeat kinase 2 (LRRK2; *PARK8*) and α-synuclein (*PARK1/PARK4*), have broad cellular and pathological roles, including several impacting mitochondrial homeostasis (24, 25). LRRK2 interacts with regulators of mitochondrial dynamics including dynamin-related protein 1 (DRP1), Rab GTPases, mitofusins, and Miro1 (26, 27, 28), and cells with *LRRK2* mutations demonstrate altered mitochondrial dynamics, reduced ATP production, and delayed mitophagy (27, 29). PD-associated α-synuclein mutations lead to mitochondrial DNA (mtDNA) damage, altered mitochondrial dynamics and respiration, and reduced mitochondrial membrane potential in cell and mouse models (30, 31, 32, 33). Furthermore, in addition to mitochondria being a direct target of α-synuclein-mediated toxicity (34, 35, 36, 37, 38), mitochondrial dysfunction may cause accumulation, phosphorylation, and aggregation of α-synuclein and therefore may contribute upstream of α-synuclein-mediated pathology (39, 40, 41, 42).

Indirect effects on mitochondria are also consequence of PD-causing mutations in genes regulating lysosomal function and the antioxidant response. Mutations in lysosomal P5 type ATPase cation transporter, ATP13A2 (encoded by *PARK9*), which cause autosomal recessive parkinsonism (Kufor–Rakeb syndrome), produce severe mitochondrial fragmentation and mtDNA lesions in fibroblasts, potentially due to cellular zinc dyshomeostasis (43, 44). Mutations in the lysosomal enzyme glucocerebrosidase (GBA), a PD risk gene, may exacerbate mitochondrial dysfunction to reduce autophagy and degradation, leading to accumulation of dysfunctional mitochondria (2, 45, 46, 47). Finally, mutation in DJ-1 (*PARK7*), a transcriptional coactivator in the cellular antioxidant response, can also cause autosomal recessive PD and is associated with a fragmented mitochondrial phenotype and increased sensitivity to mitochondrial toxins (48, 49). Additionally, distinct functions of DJ-1, including glyoxalase activity and a role in chaperoning, may also influence mitochondrial function (50).

At the most fundamental level, it remains uncertain why DA neurons are selectively vulnerable to mitochondrial dysfunction. Hypotheses include their significant bioenergetic demands and the highly polarized and branched nature of DA neurons, which creates challenges for mitochondrial trafficking through the cell. Furthermore, DA neurons are exposed to the oxidative nature of dopamine (51) and have low ETC complex I expression (52). DA neurons are particularly reliant on L-type Cav1.3 Ca^2+^ channels to facilitate continuous rhythmic pacemaking activity and therefore subject to potentially damaging effects of large Ca^2+^ transients and associated oxidative stress (53, 54, 55). Accordingly, any insult leading to even modest mitochondrial impairment is particularly neurotoxic to DA neuron populations.

Beyond genetically determined disease, mitochondrial dysfunction and reduced mitophagy are also observed in sporadic PD (6, 7, 27, 56, 57, 58). Oxidative stress and bioenergetic compromise are recognized phenotypes of PD *in vivo* and *in vitro* (56, 57, 58, 59, 60). Mitochondrial electron transport chain (ETC) complex I deficiency and increased frequency of mtDNA mutations have been identified in sporadic PD patients (60, 61), and delayed mitophagy following mitochondrial uncoupling was reported in PD patient cells (27).

### PINK1 and Parkin

**Figure 2:**
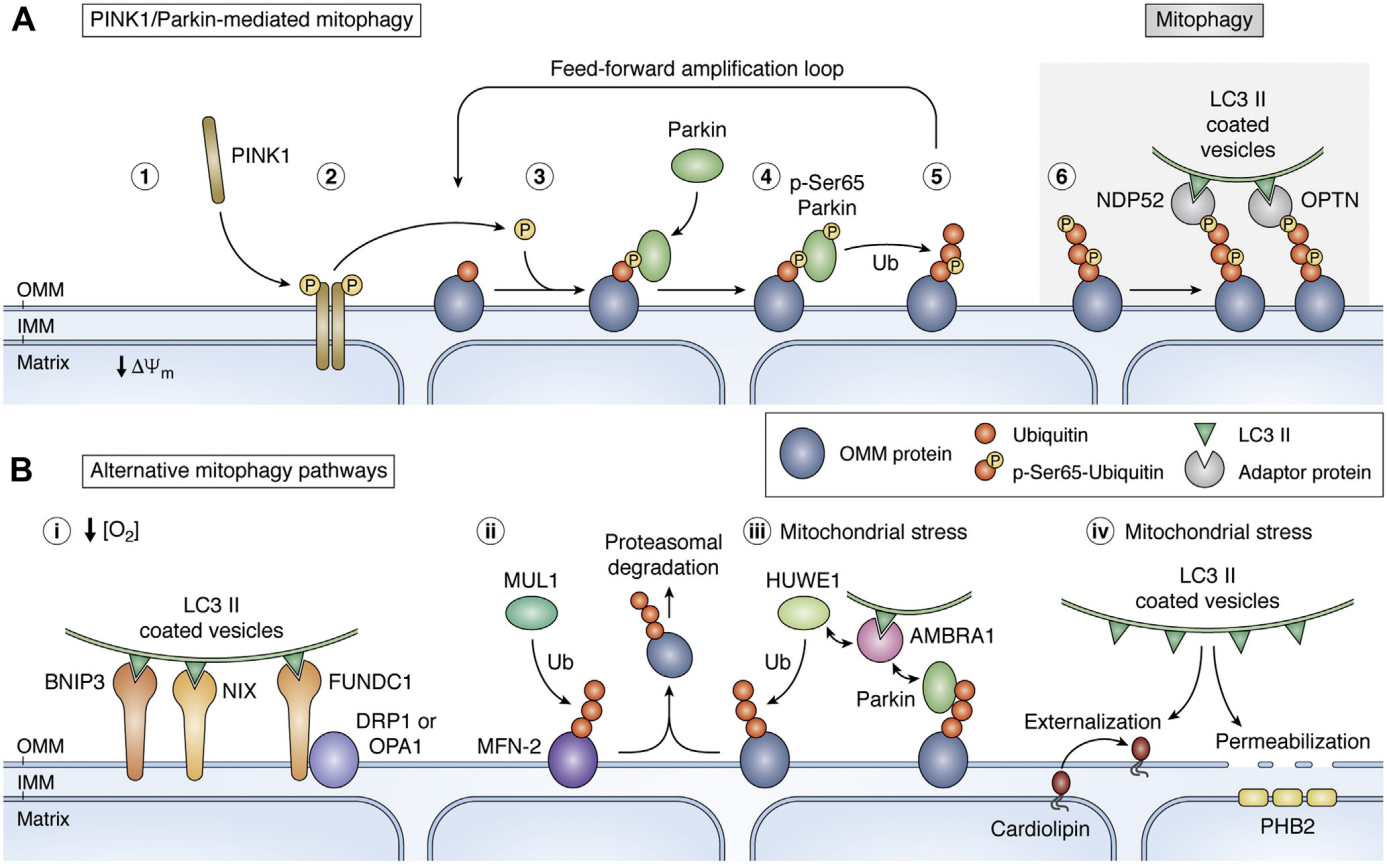
**PINK1-Parkin-dependent and independent mitophagy.** Panel A: (1). Reduction in the mitochondrial membrane potential (Δψ_m_) causes (2) PINK1 stabilization at the OMM where it dimerizes and autophosphorylates, resulting in activation. (3) Activated PINK1 phosphorylates Ub chains formed by E3-ubiquitin ligases such as March5 on OMM proteins such as TOM complex members. (4) Phosphorylated Ub chains (p-Ser65-Ub) allow the recruitment of Parkin from cytosol to OMM where it is phosphorylated at Ser65 by PINK1 and becomes fully activated. (5) Activated Parkin ubiquitinates OMM proteins generating a self-amplifying feedback loop with PINK1. (6) Adaptor proteins such as NDP52 or OPTN bring together p-Ser65-Ub chains with LC3-coated vesicles. Panel B: (i) Under hypoxic conditions, BNIP3, NIX, or FUNDC1 can bind ubiquitinated OMM proteins and recruit LC3 II-coated vesicles. (ii) MUL1 regulates ER-mitochondria contacts ubiquitinating MFN-2, resulting in its proteasomal degradation. (iii) HUWE1 and AMBRA1 interact to ubiquitinate proteins for proteasomal degradation or resulting in AMBRA1-mediated recruitment of LC3 II-coated vesicles. (iv) Cardiolipin externalization or IMM protein PHB2 exposure results in LC3 II-coated vesicle recruitment to the mitochondria.

The association between mutations in *PINK1* and *Parkin* and the development of EOPD suggest that defective mitophagy and accumulation of damaged mitochondria are key factors involved in the etiology of disease. PINK1 and Parkin act in concert within a mitochondrial quality control system that has become well characterized over the past decade or so (Fig. 2). In healthy mitochondria, the serine/threonine kinase PINK1 is targeted to mitochondria, localizing to the translocase of the outer mitochondrial membrane (TOM) complex on the OMM. PINK1 is N-terminally translocated across the OMM to the inner mitochondrial membrane (IMM) (62). Imported PINK1 is sequentially proteolytically cleaved, first by mitochondrial processing peptidase (MPP) and secondly by presenilin-associated rhomboid-like protease, PARL (63, 64). PINK1 is subsequently removed for degradation by the proteasome *via* the N-end rule, maintaining low basal levels of PINK1 protein (65). Mitochondrial injury, typically presenting as reduced mitochondrial membrane potential, prohibits import of PINK1, stabilizing the active protein on the OMM. Although mitochondrial membrane potential depolarization has long been understood as the key mechanism by which PINK1 is stabilized, further methods have been used to trigger PINK1 stabilization *in vitro* and may perhaps represent further physiological stimuli by which mitophagy is initiated. These include initiation of the mitochondrial unfolded protein response (mtUPR) using an N-terminal deletion mutant of ornithine transcarbamylase (ΔOTC) (66) and induction of spatially restricted mitochondrial oxidative damage by using a photoreactive probe called Mito-Killer Red (67). Active PINK1 autophosphorylates in *trans*, resulting in self-amplifying activity and substrate recognition (68, 69, 70). PINK1 forms a homodimer (71) and phosphorylates ubiquitin localized at the mitochondrial surface on serine 65 (Ser65) residues. March5 (also known as MITOL) is a RING finger E3-ubiquitin ligase localized to the OMM and has been proposed to catalyze formation of the initial OMM ubiquitin “seed.” The seed is proposed to serve as a substrate for PINK1-mediated phosphorylation and, subsequently, as the upstream receptor for Parkin. Thus, silencing of March5 slows Parkin recruitment to mitochondria (72).

The RING/HECT hybrid E3-ubiquitin ligase Parkin translocates from the cytosol to mitochondria and is activated *via* two mechanisms: binding to phospho-Ser65-ubiquitin and phosphorylation by PINK1 on the homologous Ser65 residue of its own ubiquitin-like (UBL) domain (69, 73, 74, 75). Binding of Parkin to phospho-Ser65-ubiquitin primes Parkin for phosphorylation within the UBL domain by PINK1. A key activating step of Parkin is the movement of phospho-UBL, its binding to RING0 and the release of the catalytic RING2 domain (76). These phosphorylation and binding events release Parkin autoinhibition to stabilize an open, active conformation capable of binding the charged E2-ubiquitin-conjugating enzyme. Active Parkin extensively ubiquitinates mitochondrial proteins (77), facilitating a feed-forward amplification loop of substrate ubiquitination, PINK1-mediated ubiquitin phosphorylation, and further Parkin recruitment to tag damaged mitochondria for removal by the autophagosome-lysosome system (74, 78, 79, 80).

Ubiquitinated proteins on the OMM act as the receptor for autophagic adaptors, including optineurin and nuclear dot protein-52 (NDP52). The subsequent recruitment and binding of the autophagy protein microtubule-associated proteins 1 light chain 3 (LC3) permit autophagosome formation (81) and lysosomal degradation. Parkin-mediated ubiquitination also targets some proteins, in particular the mitofusins, for degradation by the proteasome. Removal of these proteins early in mitophagy limits mitochondrial fusion, promoting mitochondrial fission, allowing damaged fragments of mitochondria to be sequestered from the healthy reticulum and removed (82).

### Alternative mitophagy pathways

Pathways and cellular signaling events other than PINK1-Parkin can also recruit LC3 and autophagosomes to mitochondria (Fig. 2). Mitophagy is induced in response to low oxygen (hypoxia). The OMM proteins BCL2/Adenovirus E1B 19 kDa Interacting Protein 3 (BNIP3) and Nip3-like protein X (NIX; also known as BNIP3L), members of the BCL-2 family of apoptosis regulators, have LC3-interacting domains and are upregulated during hypoxia (83). NIX is also a Parkin substrate and involved in the recruitment of autophagic adaptors to mitochondria (84).

Another OMM protein, FUN14 domain-containing protein 1 (FUNDC1), is also involved in hypoxia-induced mitophagy (Fig. 2). FUNDC1 binds LC3 independently of Parkin, altering mitochondrial dynamics during mitophagy *via* interactions with profission protein DRP1 and mitochondrial fusion protein Dynamin-like 120 kDa protein (OPA1) (85). FUNDC1 interacts with LC3 after dephosphorylation at Ser13 by serine/threonine-protein phosphatase PGAM5 and phosphorylation at Ser17 by the autophagy regulator kinase ULK1 in response to hypoxia (86). The response of FUNDC1 is fine-tuned *via* regulated ubiquitination and degradation by March5 in the initial stages of hypoxia (87).

HUWE1 is a HECT-type E3-ligase and promotes mitophagy *via* the proautophagic, LC3-interacting protein autophagy/beclin-1 regulator-1 (AMBRA1). A complex relationship exists where, upon mitochondrial stress, AMBRA1 functions as a cofactor for HUWE1, mediating both HUWE1 mitochondrial translocation and subsequent ubiquitination and proteasomal degradation of OMM proteins including MFN-2. Ubiquitination of OMM proteins is speculated to provide the signal for AMBRA1 phosphorylation at Ser1014 *via* IKKα and to promote AMBRA1-LC3B interaction and mitophagy (88). Furthermore, mitochondrial depolarization promotes a direct interaction between AMBRA1 and Parkin, activating proximal Class III PI3K, contributing to new phagophore formation (89).

MUL1 is a multifunctional RING finger mitochondrial membrane protein with both ubiquitin and small ubiquitin-like modifier (SUMO) E3-ligase activities. MUL1 functions in parallel to the PINK1–Parkin pathway to ubiquitinate and remove MFN-2, compensating when overexpressed for the mitochondrial phenotypes associated with *PINK1* and *Parkin* mutant *Drosophila* (90). Moreover, MUL1 has recently been identified as an early checkpoint to protect mitochondria from rapid degradation under mild stress. MUL1 preserves mitochondrial morphology and mitochondria–endoplasmic reticulum (ER) contact by repressing the levels of MFN-2, to maintain Ca^2+^ homeostasis and metabolism. MFN-2 accumulation leads to increased cytosolic Ca^2+^ influx, mitochondrial fragmentation, and a decrease in mitochondrial membrane potential. It is speculated that if the MUL1-MFN-2 checkpoint fails, Parkin-mediated mitophagy will be activated (91).

Finally, externalization of cardiolipin, a unique IMM phospholipid, and exposure of IMM protein Prohibitin-2 (PHB2) following mitochondrial outer membrane rupture have each been observed to recruit LC3 and act as mitophagy receptors following mitochondrial damage (92, 93) (Fig. 2). By virtue of strong genetic association, the canonical PINK1–Parkin pathway remains the primary focus for PD research. However, numerous mitochondrial quality control pathways have been described and together provide additional intervention points for potential therapies to modulate mitophagy.

## Intervention points for mitophagy-based therapeutics

PINK1-Parkin-dependent and -independent pathways provide many potential biological intervention points to enhance mitophagy. Indeed, several molecules are already available to perturb biology through inhibition or activation of a specific target (discussed in detail below). These molecules are useful to address hypothesis validity, and in some instances may provide a starting point for therapeutic development. Several additional mitophagy regulators hold potential for future therapeutic targeting.

### Protease

#### OMA1

Mitochondrial membrane depolarization induced-PINK1 import arrest is a key initiating event in mitophagy. Failure of PINK1 stabilization has been observed for several PD-linked *PINK1* variants owing to inappropriate import and cleavage by the IMM-embedded metalloprotease, OMA1. Tom7, a small accessory protein of the TOM complex, facilitates PINK1 import arrest before OMA1 recognition and has been proposed to mediate the lateral release of conformationally kinase-active PINK1 from the TOM40 channel (94). Suppression of OMA1 restores depolarization-induced import arrest of PD-related *PINK1* variants at the OMM. Additionally, mitochondrial membrane depolarization-induced PINK1 stabilization was slightly enhanced in OMA1^−/−^ cells, independent of changes in PINK1 steady-state import (94). Together these data, along with observations of an *in vivo* protective role of OMA1^−/−^ in a forebrain neuron-specific *Phb2*-deficient (*Phb2*^*NKO*^) neurodegenerative model (95), suggest inhibitors of OMA1 may be therapeutically viable.

#### USP33/VDU1

Ubiquitin chains can be removed from substrates by a family of proteins known as deubiquitinases (DUBs). USP33 is an OMM localized DUB, which antagonizes Parkin autoubiquitination mainly at Lys435. Silencing of USP33 enhances K63-linked ubiquitin chain formation on Parkin, increasing Parkin stabilization and the rate of depolarization-dependent mitochondrial translocation, accelerating mitophagy. Interestingly, USP33 is expressed at high levels in central nervous system (CNS) and PD-affected areas, including amygdala and substantia nigra (96).

#### Deubiquitinases (DUBs)

Numerous DUBs have been shown to regulate mitophagy including USP30 (discussed below), USP15 (97, 98), USP8 (99), and a splice variant of USP35 (100). These have been described elsewhere (101).

### Phosphatase

#### Protein phosphatase with EF-hand domain 2 (PPEF2)

A phosphatase antagonistic to PINK1, which dephosphorylates ubiquitin and supresses PINK1-mediated mitophagy. Silencing of PPEF2 increases phospho-Ser65-ubiquitin and enhances basal and stress-induced mitophagy both dependent and independent of Parkin. Interestingly, numerous proteins have been identified as being inversely regulated by PPEF2 and PINK1. These observations point to potential roles in mitochondrial biogenesis, regulation of mitophagy in cells and tissues with low Parkin expression, and as an antiapoptotic phosphatase (102). Notably, phosphorylated ubiquitin linkages have greater resistance to DUB-mediated cleavage, suggesting that dephosphorylation of ubiquitin may be a critical regulator in controlling rates of mitophagy (103).

#### PTEN-L

A translational variant of PTEN, localized to the OMM and cytosol. Comparable with PPEF2, PTEN-L antagonizes PINK1-mediated phosphorylation of ubiquitin, reducing Parkin translocation and relief of autoinhibition and thereby suppressing mitophagy. PTEN-L decreases abundance of phospho-Ser65-Parkin; however, it is unclear if PTEN-L dephosphorylates Parkin directly or whether the reduction is secondary to reduced mitochondrial translocation and proximity to PINK1 (104).

## Mitophagy reporter assay systems

Several mitophagy reporters have been developed to exploit the pH differential between cellular compartments and discrete organelles to discriminate stages of mitophagy. These have been effectively used to study mitophagy *in vitro* and *in vivo*.

### Mt-keima

mt-Keima utilizes unique fluorescent properties of the coral-derived protein Keima, artificially targeted to the mitochondrial matrix using the COX8A mitochondrial targeting sequence (MTS) (105). Keima has a single, pH-independent emission peak at 620 nm but a pH-dependent bimodal excitation. The excitation maximum at 440 nm in slightly alkali environments (the mitochondria) shifts to 586 nm in the acidic environment of the lysosome. Ratiometric analysis of 586 nm: 440 nm fluorescence intensity yields a “mitophagy index,” describing the relative proportion of mitochondria within acidic lysosomes (pH 4.5) to healthy mitochondria with normal matrix pH (pH 8) residing within the cytoplasm (105, 106). Several mechanistic and biological properties however limit the use of mt-Keima. Keima is incompatible with immunohistochemical fixation, which dissipates proton/pH gradients, preventing delineation of mitophagy index within specific cell populations. Additionally, though studies have demonstrated that Keima is relatively insensitive to proteolytic degradation, the fate of lysosomal mt-Keima protein remains ill-defined (105). The partial overlap of the 440:586 nm bimodal excitation ranges of Keima in different pH environments may also complicate ratiometric analysis (106).

### Mito-QC (mCherry-GFP-FIS1(aa.101–152))

Mito-QC exploits the pH-sensitive quenching of green fluorescent protein (GFP) in acidic environments. The targeting sequence from FIS1 directs a tandem GFP-mCherry protein to the OMM. Within the cytosol, mito-QC fluoresces both red and green, however, upon delivery to the lysosome GFP is quenched, allowing analysis of mCherry puncta as index of mitophagy (107). However, mito-QC is cytoplasmic facing, making it open to extraction and clearance by OMM proteasome-dependent pathways. Indeed recently, proteasome-sensitive Parkin-dependent clearance of mito-QC following mitochondrial uncoupling has been observed (108). Further, much like mt-Keima, constitutive expression over the course of mouse development may produce basal signal of unknown specificity, which may mask subtle changes in mitophagic signaling. This basal signal may also reflect tissue-specific differences in lysosomal activity and clearance. Notably, inducible expression of mito-QC reduced Parkin-independent signals (108).

### Mito-SRAI (mitochondrial matrix targeted signal-retaining autophagy indicator)

Mito-SRAI is a tandem YPet-afCFP (a.k.a. TOLLES: TOLerance of Lysosomal EnvironmentS) construct. Acid sensitivity of YPet allows distinction of TOLLES-positive puncta in acid compartments, indicating mitophagy (108). Unlike mito-QC, initial characterization of mito-SRAI *in vitro* demonstrates insensitivity to proteasomal clearance, and unlike mt-Keima, mito-SRAI is amenable to fixation.

## Pharmacological enhancement of mitophagy: from tool molecules to potential therapeutics

The relationship between mitochondrial dysfunction and PD suggests that improving the efficiency of mitochondrial clearance by mitophagy may be a disease-modifying strategy for PD (Table 1). Increased understanding of mitophagy pathways has led to the identification of potential therapeutic targets and intervention points to positively modulate mitophagy. To date, several small molecules and natural compounds targeting mitophagy have been identified using target-based, *in silico*, or phenotypic screening strategies and, encouragingly, have subsequently demonstrated neuroprotection in PD models (Table 1).

**Table 1:** Structure and experimental summary of molecules enhancing mitophagy

Compound	Structure	Target	Mechanism of action	Read out	References
AUTAC4		TSPO	PINK1–Parkin-independent mitophagy	ICC: LC3 II puncta colocalization with mito-EGFP-HT or K63-linked Ub, Mito-Rosella dye, MtPhagy dye	(137)
BC1464		FBXO7	PINK1-dependent mitophagy	WB: Phosphorylation of Ubiquitin	(13)
Compound 3		Miro1	Unknown mechanism, potentially through PINK1	WB: MFN-2, VDAC, LRRK2, Parkin ICC: TOM20, ATP5β	(128)
Deferiprone		Iron	PINK1-independent mitophagy	WB: MFN-2, HSP60, TIMM50, Omi ICC: mCherry-GFP-FIS1(aa.101–152) IHC: mt-Keima	(107)
FT385		USP30	PINK1–Parkin-dependent mitophagy	WB: Ub-TOM20/TOM20, PINK1 ICC: mCherry-GFP- FIS1(aa.101–152)	(125)
Gemcitabine		Not reported	MUL1, PINK1-dependent mitophagy	ICC: mt-Keima, high-content image analysis	(136)
GYY4137		Pleiotropic/Keap1	PINK1–Parkin dependent mitophagy	Biochemical: Parkin E3-ubiquitin ligase activity	(233)
Ivermectin	Mixture of 2 isomers	TRAF2 proposed	PINK1–Parkin-independent mitophagy	WB: TOM20 ICC: LC3 II and mCherry-colocalization with TOM20 Electron microscopy: mitophagosome formation	(149)
Kinetin		PINK1	PINK1-dependent mitophagy	ICC: Parkin–mitochondria colocalization	(109, 110, 112)
Kinetin Riboside		PINK1	PINK1-dependent mitophagy	ICC: Parkin–mitochondria colocalization	(110)
Nicotinamide		NAD(+)-precursor	SIRT1-PCG1α-dependent mitophagy	WB: LC3, PINK1 ICC: LC3 II, mitochondrial morphology, mitophagy dye	(144, 145)
Nilotinib		c-Abl	Parkin-dependent mitophagy	WB: LC3, phospho-Ser65-Ub ICC: phospho-c-Abl and Parkin colocalization	(121)
p62-mediated mitophagy inducer (PMI)		Nrf2	PINK1-independent mitophagy p62-dependent mitophagy	ICC: colocalization Parkin, ATP synthase β subunit	(126)
MWP00839		Not reported	Unknown mechanism	ICC: mito-Timer high-content image analysis	(133)
SPB08007					
SR3677		ROCK2	PINK1-dependent mitophagy	WB: MFN-2, VDAC1 ICC: Parkin- mito-dsRed colocalization, mito-QC	(129)
Y27632		ROCK1/2	PINK1-dependent mitophagy	ICC: Parkin–mitochondria colocalization	
Sulforaphane		Keap1	p62-dependent mitophagy	ICC: p62/SQSTM1, LC3	(126)
T0466		Not reported	PINK1–Parkin dependent mitophagy	ICC: High-content image analysis, Luciferase tagged MFN-2	(132)
T-271		Not reported	Parkin-dependent mitophagy	ICC: High-content image analysis, mito-SRAI signal	(108)
USP30i		USP30	PINK1-dependent mitophagy	WB: Ub-TOM20	(124)
Urolithin A		Not reported	Unknown mechanism, potentially through PINK1	WB: PINK1, Parkin, pTBK, pULK, p62, LC3 I/II, Ub ICC: GFP-DsRed, MitoRosella, TOM20-LAMP2 colocalization, Mitophagy Dye, mito-GFP, LC3B-GFP, mRFP-GFP-LC3B Electron microscopy	(139, 140)

aa, amino acids; CFP, cyan fluorescent protein; ICC, immunocytochemistry; Ub, ubiquitin; WB, western blot.

### Target-based drug discovery strategies for mitophagy enhancement

PINK1 is a mitochondrially localized serine/threonine kinase with a direct genetic relationship with PD (3), as described above. Disease-causing mutations in the kinase domain and C-terminal noncatalytic region of PINK1 suppress catalytic activity, leading to the hypothesis that restoration of kinase activity may have disease-modifying effects in PD (109). Indeed, PINK1 can induce mitophagy even in the absence of Parkin (81), suggesting PINK1 is an important and potentially druggable intervention point for therapeutic development. PINK1 is unique among kinases in its ability to accept the neo-substrate kinetin triphosphate (KTP; N6 furfuryl ATP) with greater catalytic efficiency than ATP, creating opportunities for drug development (109) (Table 1). KTP is produced by consecutive metabolic steps from kinetin or kinetin riboside (KR), once internalized into the cell (109). However, the efficiency by which cells convert kinetin and KR to KTP is low. KR monophosphate also has poor cellular stability. To overcome these limitations, KR ProTides (PROdrug + nucleoTIDE) have been developed to deliver KR into the cell (110) (Table 1). Cellular studies have shown that once converted to its active form (KTP), kinetin administration can enhance PINK1 activity. Kinetin treatment increases Parkin translocation to mitochondria and reduces mitochondrial motility in neuronal axons, critical steps in removal of damaged mitochondria by mitophagy (109). Furthermore, kinetin prevents cleavage of BCL-XL to its proapoptotic form through increasing PINK1-mediated phosphorylation of BCL-XL at Ser62 (109, 111). Interestingly, *in vivo* pharmacokinetic studies have demonstrated that kinetin crosses the blood–brain barrier (BBB) and is well tolerated in humans (112, 113).

Genetic rationale supports targeting Parkin for drug development (11) although complex structural biology, autoinhibition, and promiscuity among target proteins create challenges. Parkin ubiquitinates a large collection of functionally diverse proteins and is believed to have little or no requirement for defined consensus sequences to determine substrate specificity (77). Instead, specific PTMs or substrate conformation may be required (72). Compelling structural studies have identified both naturally occurring and artificially designed activating mutations in Parkin (114). Activating Parkin mutations can rescue phospho-dead and UBL-domain-deleted Parkin (115) as well as many pathogenic PD mutations (116). These data collectively provide proof-of-concept that recessive Parkin mutations in PD can be rescued and highlights the potential that rational drug design may produce pharmacological agents that mimic conformational changes associated with activating mutations. Furthermore, Gladkova *et al.* (117) identified a small conserved helix in the Parkin UBL-RING0 linker, known as the activation element (ACT), which contributes to catalytic RING2 domain release by mimicking RING2 interactions in the RING0 domain and may potentially serve as a scaffold for the creation of a small-molecule Parkin activating compounds. Additionally, although peer-reviewed research is not available, several activators of Parkin have been described in the patent literature (US 2016/0160205A1 and WO 2018/023029). These compounds provide the first evidence of direct Parkin modulation.

An alternative and indirect strategy is to modulate Parkin activity *via* endogenous regulators such as c-Abl, a tyrosine kinase with a prominent role in neurons. c-Abl regulates Parkin by phosphorylation at Tyr143, resulting in decreased Parkin activity and reduced mitophagy. Interestingly, increased levels of phosphorylated, active c-Abl have been found in PD brains (118, 119). Nilotinib, a c-Abl inhibitor, is currently used to treat chronic myelogenous leukaemia (CML) (Table 1). Nilotinib prevents α-synuclein accumulation and dopaminergic cell loss in an *in vivo* model of PD (120), and has been investigated in PD clinical trials (NCT02281474, NCT02954978). As c-Abl negatively regulates Parkin, it has been proposed that some of nilotinib’s protective effects are related to modulation of Parkin-dependent pathways (121). Nonetheless, further studies are needed to determine whether Parkin activation by nilotinib can induce mitophagy as a protective approach in neurodegeneration (Table 1). Some authors have highlighted nilotinib inhibition of kinases other than c-Abl, suggesting that it may achieve neuroprotective effects *via* these other pathways (122).

Ubiquitination of mitochondrial proteins is tightly regulated at multiple different levels. Ubiquitin specific protease 30 (USP30) is a mitochondrially localized DUB, hypothesized to oppose Parkin-mediated mitophagy by removing poly-ubiquitin chains from damaged mitochondria (123). Loss of USP30 enhances both stress-induced and basal mitophagy. USP30 has lower activity against phosphorylated ubiquitin linkages, therefore potentially acting upstream or independently of PINK1 (103, 123). This provokes the hypothesis that USP30 may act on the initial mitochondrial ubiquitin “seeds” before PINK1–Parkin activation, defining a threshold for mitophagy initiation, and therefore making it an attractive target for modulating mitophagy in PD.

Two recent studies described potent inhibitors of USP30 (Table 1) (124, 125). FT3967385 (FT385) was used as a tool to study the impact of USP30 inhibition on the total cellular ubiquitinome, identifying only subtle effects overall but a large impact on ubiquitination of mitochondrial proteins such as voltage-dependent anion channel (VDAC) and TOM components. A significant increase in phospho-Ser65-ubiquitin and mitophagy was also observed (125). The authors proposed a model in which USP30 regulates the ubiquitin chains available for PINK1 phosphorylation following mitochondrial depolarization. The study concluded that USP30 plays a key role in regulating activities of PINK1 and Parkin, suggesting USP30 inhibition as a viable strategy to induce mitophagy (125).

A second study shows that USP30 also antagonizes the effect of E3-ubiquitin ligases other than Parkin to regulate distinct mitochondrial functions. Using a newly reported USP30 inhibitor, USP30i, as a tool, the authors dissected the mechanisms by which USP30 works together with March5 to regulate import of proteins into the mitochondrial matrix (124). In agreement with the previous study, Phu *et al.* (124) demonstrated that USP30 regulates TOM complex component ubiquitination, which may serve as a potential ubiquitin seed for PINK1-Parkin function. Importantly, USP30 inhibition enhanced ubiquitin phosphorylation and mitophagy even in the absence of Parkin, placing USP30 upstream of Parkin. This study supports USP30 as a possible invention strategy for mitophagy enhancement even in the context of PINK1 or Parkin dysfunction.

Expression of several autophagy adaptor proteins involved in mitophagy, including p62, is positively regulated by the transcription factor, nuclear factor erythroid 2-related factor 2 (Nrf2). Based on this mechanism, an Nrf2 pharmacological inducer, known as p62-mediated mitophagy inducer (PMI), has been developed (Table 1) (126). PMI stabilizes Nrf2, resulting in higher p62 expression and induction of mitophagy. In contrast with previously reported compounds exploiting this mechanism such as sulforaphane (Table 1), PMI does not contain a covalent-binding motif and may have less toxic potential (126).

### Targeted *in silico* screening to identify novel enhancers of mitophagy

Mitochondrial motility is critical in neuronal physiology. Modulating mitochondrial dynamics to facilitate mitophagy might be a promising approach, especially in the context of highly polarized neurons where mitochondrial trafficking plays an important role in cell physiology. Miro1 is an OMM GTPase participating in mitochondrial trafficking and impairment of Miro1 clearance is associated with both familial and sporadic PD (27). Ubiquitination and degradation of Miro1 is a critical step in mitophagy initiation (127). Recent efforts to target Miro1 in the context of PD have led to the characterization of Compound 3 as a mitophagy inducer (Table 1) (128). For compound identification, machine learning was applied to predict the docking capacity between molecules and protein structures based on available knowledge. This approach was subsequently used to virtually screen for compounds able to bind C-terminal region of Miro1, the region being the minimal element required to be recognized and ubiquitination by Parkin. Starting from a large library of over 6 million commercially available molecules, the study narrowed down the drug candidates to 11 promising compounds, which were tested *in vitro*, four of which showed activity. Further characterization revealed that Compound 3 (Table 1) reduced Miro1 levels after mitochondrial depolarization in patient-derived PD fibroblasts, showing a neuroprotective effect, both *in vivo* and *in vitro* models (128).

Recently, *in silico* screening identified a novel inhibitor (BC1464) of FBXO7, an E3-ligase complex adaptor protein (described above). Interestingly, the authors identified that FBXO7 can target PINK1 for degradation (13). The substrate-binding FP-domain within FBXO7 was interrogated as a target region for small molecule interactions. Starting from a virtual library of 3 million compounds, docking experiments on a three-dimensional (3D) model of the FP-domain allowed the authors to narrow down to 20 hit compounds to perform further biochemical studies. From these compounds, BC1464 increased PINK1 levels by preventing PINK1-FBXO7 interaction and subsequent PINK1 degradation, without loss of mitochondrial membrane potential. Importantly, BC1464 prevented MPP+-induced cell death *in vitro* and protected human fibroblasts derived from PD patients carrying *LRRK2* mutations exposed to 6-OHDA (13). Together, these studies highlight the impact of *in silico* screening and advances in machine learning, processing power, and computational biology to help yield promising hit molecules.

### Phenotypic screening strategies for mitophagy enhancement

PD is a complex multifactorial disease and the complex nature has hampered PD drug development. One approach with potential to overcome previous methodological limitations is the application of phenotypic screening. Phenotypic screens aim to identify hit compounds by measuring the effect of the molecules on a disease-related phenotype. This screening paradigm provides an unbiased approach as it does not require a specific drug target. Output from a phenotypic assay should both reflect and be able to predict success in modulating the physiological endpoint of interest. This assumes a shared mechanistic basis between bioassay endpoint and disease phenotype, which is necessary to create robust predictive power for therapeutic development. Together these elements provide the ability to identify novel small molecules and novel biological modifiers of mitophagy.

Moskal and coworkers (129) recently applied a high-throughput screen (HTS), employing Parkin translocation into the mitochondria as a readout of mitophagy activation. The authors stably expressed GFP-tagged Parkin in HEK293 cells and, using a machine-learning approach for image analysis, identified hit compounds enhancing GFP-Parkin translocation. The study identified SR3677, a Rho-associated coiled-coil containing protein kinase 2 (ROCK-2) inhibitor, as a hit compound. Further characterization suggested that SR3677 enhances Parkin-mediated mitophagy, potentially *via* increased recruitment and activity of Hexokinase II, promoting Parkin translocation (129). SR3677 was found to be protective in an *in vivo* model of Parkinsonism. The authors caution, however, that the effect of ROCK inhibition may be *via* destabilization of the actin cytoskeleton and encapsulation of depolarized mitochondria by F-actin cages, hence the mechanism requires confirmation (129).

Another recent HTS assessed degradation of luciferase-tagged mitofusin-1 (MFN-1) as a readout of Parkin activity. MFN-1 is an OMM GTPase mediating mitochondrial fusion (130) and has a critical role in mitophagy. On damaged mitochondria PINK1 phosphorylates MFN-1, contributing to recruitment of Parkin, which in turns ubiquitinates MFN-1, resulting in MFN-1 degradation and mitochondrial fragmentation (131). Here, mitophagy was assessed in HeLa cells stably transfected with luciferase-tagged MFN-1, in the presence and absence of Parkin expression to determine Parkin dependence. Using this approach two new molecules, T0466 and T0467, were identified (Table 1) from a library of ∼45,000 compounds. Both compounds were able to induce Parkin translocation to the mitochondria without loss of mitochondrial membrane potential or toxicity in DA neurons. Additionally, both compounds improve motor defects in the *PINK1* knockout mitochondrial degeneration *Drosophila* model (132).

Mitochondrial turnover is vital to maintain long-term mitochondrial capacity. As such, mitochondrial biogenesis is intimately linked to mitophagy. Considering this, a recent study using high-content analysis of Mito-Timer reporter was developed (133). Mito-Timer is based on a mitochondrially targeted dsRed1-E5, a mutated form of dsRed fluorescent protein. dsRed1-E5 evolves from green to red fluorescence in a time of 18 to 20 h, allowing measurement of mitochondrial age and turnover (134, 135). Using the time-dependent fluorescent properties of Mito-Timer, a study found two new mitophagy inducers, SPB08007 and MWP00839 (Table 1), from a library of ∼15,000 molecules. Both compounds were able to increase mitochondrial turnover by stimulating mitophagy without causing reduction of mitochondrial membrane potential or increased superoxide formation (133).

HTS relies on the availability of robust, specific readouts to measure the process of interest. Mito-SRAI has been recently described as a new mitophagy reporter. Using a human glioblastoma H4 cell line stably expressing Mito-SRAI, the authors successfully applied this new probe to a large-scale high-content image analysis approach to identify novel mitophagy enhancers within a library of ∼76,000 compounds (108). The screen focused on compounds inducing Parkin-dependent, Bafilomycin A-1 sensitive mitophagy and does not affect mitochondrial membrane potential, identifying T-271 as a novel mitophagy inducer (108), while also validating a novel mitophagy reporter system.

Finally, using the mt-Keima reporter in HeLa cells and the LOPAC1280 chemical library, several mitophagy-enhancing compounds have been identified. One compound, the anticancer drug gemcitabine, was identified as inducing mitophagy independent of Parkin (Table 1) but dependent on MUL1. Gemcitabine caused the stabilization of PINK1 without reducing mitochondrial membrane potential (136).

Together, these studies have demonstrated the importance of high-throughput/high-content phenotypic screening and the development of innovative image analysis pipelines in the identification of novel compounds. The compounds identified are not only interesting therapeutic candidates, targeting different components of the mitophagy machinery without causing toxicity, but also provide insights into the molecular biology of mitophagy in both physiological and pathological context. However, these studies often lack information about the specific target of the newly identified molecules, and subsequent deconvolution is necessary to further understand the precise mechanism modulating the cellular functions.

### Novel modalities to enhance mitophagy: targeted protein degradation using AUTACs

PINK1-Parkin activation generates a feed-forward loop in which poly-ubiquitin chains accumulate on OMM proteins, targeting mitochondria for degradation *via* autophagosomes (80). Based on this system, autophagy targeting chimera 4 (AUTAC4) has been recently described (Table 1). AUTAC4 is a bivalent chimera combining a 2-phenylindole derivative, a ligand of the OMM translocator protein (18 kDa) TSPO, and a guanine tag, to induce Lys63-linked poly-ubiquitination on the OMM, separated by a linker. S-guanylation has been identified as a moiety that can independently trigger cargo-selective autophagy (137). This chemical arrangement together results in targeting of mitochondria for mitophagic degradation. Importantly, AUTAC4 specifically triggered mitophagy only with depolarized and fragmented mitochondria, as after uncoupler treatment, in HeLa cells, independently of PINK1 and Parkin. It has been hypothesized that intact mitochondria and those not excised from the mitochondrial network are too large to be degraded by a phagosome (137), leading to the specificity of AUTAC4 for small, damaged mitochondrial fragments. Altogether, AUTAC4 and modulation of poly-ubiquitin chain formation are promising approaches in developing novel mitophagy inducers. However, limitations in physicochemical properties of these molecules may affect their therapeutic development potential (138).

### Small molecule enhancers of mitophagy with complex or undefined mechanism

There are an increasing number of promising mitophagy inducers with a good safety profile but without a fully elucidated mechanism of action. The elegantin Urolithin A (UA) has been extensively studied in clinical trials to prevent aging associated changes. UA has been observed to increase life span and cognition in *C. elegans* as well as improve muscular function in rodents (139). The mechanism of action is not fully understood, although beneficial effects of UA are believed, at least in part, to be due to induction of PINK1-Parkin dependent mitophagy (139, 140) (Table 1). Nicotinamide, a precursor to nicotinamide adenine dinucleotide (NAD^+^), has been extensively studied in clinical trials for age-related diseases including PD and Alzheimer's disease (NCT03568968, NCT03816020). The therapeutic potential of nicotinamide has been associated with the NAD^+^-dependent deacetylase, SIRT1 (141, 142). SIRT1 function is dependent on the intracellular NAD^+^ levels, and a high ratio of NAD^+^/NADH is associated with longevity and enhanced mitochondrial metabolism (143). Nicotinamide has been proposed to induce mitophagy *via* SIRT1 activation, resulting in mitochondrial clearance independent of mitochondrial membrane potential depolarization (144, 145) (Table 1). Deferiprone, an iron chelator, has undergone Phase II clinical trials to evaluate this mechanism to reduce oxidative stress in the SNc of PD patients (NCT02655315, NCT01539837, NCT00943748, NCT02728843). Clinical data has emerged, demonstrating reduction in iron content in specific brain areas, a trend for improvement in motor function and improved quality of life without significant side effects (146, 147). Iron chelation can trigger mitophagy *in vitro* and *in vivo*, independently of PINK1 and mitochondrial membrane potential dissipation although the exact mechanism needs to be further elucidated (107, 148) (Table 1). Finally, the anthelmintic drug ivermectin has also recently been described as potent inducer of ubiquitin-dependent mitophagy (Table 1), working *via* a mechanism independent of PINK1 and Parkin potentially involving other E3-ligases, TRAF2, cIAP1, and cIAP2 (149) (Table 1).

In summary, many compounds are available to perturb mitophagy and several show efficacy in disease models. One caveat of many of the studies described above is the use of mitochondrial toxins to induce mitophagy. These compounds trigger mitophagy *via* mitochondrial damage, either by inhibition of the respiratory chain, ROS generation (as in the case of rotenone or antimycin A), or directly collapsing mitochondrial membrane potential (such as the ionophores CCCP, FCCP, and valinomycin). These compounds have proven invaluable in understanding the cell biology around mitophagy. However, the mechanisms to induce mitophagy in the experimental systems must be considered in wider implications of the results. A second caveat is the use of Parkin overexpression. Expressing high levels of the E3-ligase Parkin may result in supraphysiological ubiquitination of proteins. Reintroduction of Parkin into Parkin-negative cells has been used to good effect in determining compound mechanism; however, expression levels should, ideally, be carefully titrated to represent endogenous concentrations within related cell types. Nevertheless, good progress has been made over the past 5 years and therapeutic promise of this pathway continues to develop.

Selection of an appropriate intervention point for mitophagy accelerating therapeutics must also consider the growing evidence of autophagy-independent, lysosome-dependent mitochondrial degradation through PINK1 or Parkin, outside the canonical mitophagy pathway (150). PINK1 and Parkin have been associated with formation of mitochondrially derived vesicles (MDVs), which traffic damaged mitochondrial components directly to the lysosome as a response to oxidative stress (151, 152, 153). Furthermore, damaged respiratory chain components have been observed to be selectively eliminated from mitochondria in a PINK1- and Parkin-dependent manner, distinct from *en masse* mitochondrial degradation (150). These data suggest that accelerating the autophagic steps of PINK1–Parkin signaling may not be the only route to therapeutic benefit. Appropriate *in vitro* models and endpoints may allow identification and targeting of different aspects of the PINK1–Parkin system, though careful validation will be necessary.

## *In vivo* models for proof-of-mechanism and proof-of-concept studies

To date, only a limited number of mitophagy-enhancing compounds have been assessed *in vivo*. However, several *in vivo* models exist for investigating the contribution of PINK1 and Parkin to mitophagy and disease. These models may provide a platform to assess future pharmacological enhancers of mitophagy.

### Pharmacodynamic modeling using mitophagy reporter mice

*In vivo* models are required to enable analysis of the spatiotemporal dynamics of mitophagy as a pharmacodynamic endpoint for testing novel therapeutic candidates. To that end, mice ubiquitously expressing the mt-Keima reporter at the Hip11 locus and mito-QC at the Rosa26 locus have been generated (105, 154). A small number of studies using either mt-Keima or mito-QC “mitophagy reporter” models have identified pervasive basal mitophagy, often with significant heterogeneity even within the same tissue type (Table 2) (106, 154). Numerous metabolic and pathogenic insults, including hypoxia, expression of mutant huntingtin (HTT), and accumulation of mtDNA mutations have been demonstrated to perturb mitophagy (106, 155). Interestingly, global knockout of the key mitophagy gene *Pink1* failed to modulate basal mitophagy in any tissue analyzed in mice (mito-QC; *Pink1*^−/−^ mouse) (155). However, following exhaustive exercise-induced metabolic stress, PINK1-dependent mitophagy was observed in the heart, and a reduced mt-Keima signal observed following *Pink1* knockout (mt-Keima; *Pink1*^−/−^ mouse) (156). Recently, a novel FRET mitophagy reporter has been described: Mito-SRAI (108). Although full *in vivo* analysis has yet to be completed, AAV-expressed mito-SRAI within SNc has been assessed in mice. Unilateral 6-hydroxydopamine (6-OHDA) administration produced a mitophagy signal in numerous mito-SRAI infected neurons. Interestingly despite loss of midbrain DA neurons following administration of 6-OHDA, the mitophagy signal originated only from tyrosine hydroxylase (TH)-negative (non-DA) neurons (108).

**Table 2:** *In vivo* mitophagy reporters and mitochondrial dysfunction-induced neurodegeneration models

*In vivo* model	Promoter and expression pattern	Model description	Characteristics and phenotype	References
**Mitophagy Reporter Models**				
mt-Keima	Hip11 locus (ubiquitous expression) CAG-promoter driven expression	Mitophagy reporter Not amenable to chemical fixation	**Mitophagy phenotype [whole body]:** considerable heterogeneity in mitophagy within the same tissue. Low levels of mitophagy in the thymus, high in the heart. **Mitophagy phenotype [brain]:** high anatomic variation. Cortex, striatum, and substantia nigra exhibit modest levels of basal mitophagy. Mitophagy greater in dentate gyrus, lateral ventricle, and Purkinje cell layer within the cerebellum. Reduced mitophagy in dentate gyrus of aged mice (3 *versus* 21 months; 70% reduction). **Pathological insult:** age-related decline in mitophagy in dentate gyrus. Expression of mutant human Huntingtin’s transgene reduced mitophagy in dentate gyrus. Low oxygen (10% oxygen) significantly increased hepatic mitophagy.	(105)
Mito-QC (mCherry-GFP- FIS1(aa.101–152)	Rosa26 locus (ubiquitous expression) CAG-promoter driven expression	Mitophagy reporter Amenable to chemical fixation	**Mitophagy phenotype [whole body]:** considerable heterogeneity of mitophagy within the same tissue. High levels within cortex of adult kidney, differential mitophagy between proximal (high mitophagy) and distal (low mitophagy) convoluted tubules within kidney. **Mitophagy phenotype [brain]:** pronounced mito-lysosomes within Purkinje cell layer. Significant mitochondrial turnover in the Purkinje somata. **Pathological insult:** no change in mitophagy in any tissue analyzed with *Pink1* knock-out.	(154, 155)
mt-SRAI-CL1-PEST	AAV- expression into right SNc	Mitophagy reporter Amenable to chemical fixation	**Mitophagy phenotype [Brain]:** numerous infected neurons positive for mitophagy signal in 6-OHDA-injected mice (same route as for viral infection); mitophagy signal in TH-negative (non-DA) neurons only.	(108)
**Genetic Neurodegenerative Models of Mitochondrial Origin**				
**Disruption of mtDNA Homeostasis**				
Mutator (POLγA^D257A^)	PolγA locus; ubiquitous expression	Homozygous knock-in mutant of PolγA (nucleus-encoded catalytic subunit of mtDNA polymerase) D257 A mutation causes loss of 3′-5′ exonuclease activity necessary for proof-reading newly synthesized mtDNA	**Aging phenotype:** decreased lifespan and premature onset of age-associated phenotypes (weight loss, reduced subcutaneous fat, alopecia, kyphosis, osteoporosis, anemia, reduced fertility, and cardiac hypertrophy). **Neuronal phenotype:** No neurodegeneration up to 12 months. Intact nigralstriatal pathway, no astrogliosis. POLγA^D257A^ (Mutator); Parkin^−/−^: large reduction in TH-positive (DA) neurons in midbrain. Reduced striatal dopamine, decreased DA metabolites. L-DOPA responsive motor phenotype. No neuroinflammation or Lewy body formation. **Mitochondrial phenotype:** 3-5x increase in mtDNA point mutations, increased mtDNA deletions. Reduced mtDNA copy number. Random point mutations in genes for respiratory chain subunits. Increased apoptosis. Little age-related decline in cardiac mitochondrial fitness. Increased megamitochondria in aged hearts (6 months). **Mitophagy phenotype:** Increased phospho-Ser65-ubiquitin in cortex (not liver), increased hepatic mitophagy (POLγA^D257A^; mt-Keima), reduced Parkin protein expression. POLγA^D257A^ (Mutator); Parkin^−/−^mice: strong inflammatory phenotype (high serum IL-6, IFNβ1, TNF, IL-1β, CCL2, IL-12(p70), IL-13, IL-17, CXCL1 and CCL4). No change in mtDNA mutation frequency compared with POLγA^D257A^; Parkin^+/+^, but reduced mtDNA pathogenicity. Reduced ETC complex activity (complex I and III). POLγA^D257A^ (Mutator); Parkin-Tg and POLγA^D257A^ (Mutator); Parkin^−/−^: Parkin fails to prevent accelerated cardiac aging.	(105, 156, 159, 160, 161, 162, 234)
mitoPARK (DAT-cre x Tfam^loxP^)	DAT promoter: DA neuron expression Homozygous deletion of mitochondrial transcription factor A (*Tfam*)	TFAM knockout in midbrain DA neurons TFAM knockout leads to mtDNA depletion and abolishes mtDNA expression.	**Neuronal phenotype:** adult onset of slowly progressive motor impairment, loss of TH-positive neurons and TH-positive terminals in striatum; depletion of nigral and striatal dopamine, age-dependent reduction in soma size and neurite branching in DA neurons. Loss of dopamine in olfactory bulb, intraneuronal inclusions, cognitive dysfunction (preceding motor dysfunction). Gastrointestinal dysfunction, gut inflammation, and gut-microbiome changes. Age-dependent L-DOPA responsive motor phenotype. **Mitochondrial phenotype:** Severe respiratory chain deficiency, reduced cytochrome oxidase subunit I expression and activity in midbrain DA neurons, fragmentation, large mitochondrial aggregates. Reduced distal axonal mitochondria [dysfunctional axonal mitochondrial transport]. **Mitophagy phenotype:** Endogenous Parkin recruitment not detected [potential technical limitations or low expression]. Tfam^loxP/loxP^; DAT-cre; AAV-Parkin-mCherry: no Parkin colocalization with mitochondria. Tfam^loxP/loxP^; DAT-cre; Parkin^−/−^: no Parkin-dependent effect on mitochondrial aggregates, mitochondrial morphology, locomotion, or TH-positive cell loss in SNc.	(163, 164, 166, 235, 236)
PD-mito-PstI	DAT promoter-driven tetracycline transactivator protein (tTA) Inducible mito-PstI exclusively in DA neurons	Expression of tetracycline-sensitive mitochondria-targeted restriction enzyme, *Pst*I, in DA neurons Mitochondrial matrix localization—COX8A MTS Mito-targeted restriction enzyme damages mtDNA in DA neurons	**Neuronal phenotype:** progressive degeneration of the DA population within SNc, striatal dopamine depletion, age-dependent loss of TH-positive neurons, L-DOPA reversible motor deficit. Locomotor deficits precede TH-positive cell loss. Absence of inclusions. Motor phenotypes initially arise from a striatal dysfunction. **Mitochondrial phenotype:** double strand breaks in mtDNA, mtDNA depletion, mtDNA deletions, ETC dysfunction. **Mitophagy phenotype:** PD-mito-PstI; Parkin^−/−^: mild acceleration, but no worsening of motor dysfunction and neuronal degeneration.	(167, 168)
Twinkle-duplication (Twinkle-Tg)	Transgenic expression of Twinkle (in-frame duplication of aa. 353–365) TH promoter: DA neuron expression 4x Twinkle [mRNA] increase in Twinkle-Tg	In-frame duplication of the mitochondrial DNA helicase, Twinkle Disruption of mtDNA replication	**Neuronal phenotype:** motor impairment, decreased TH-positive neurons, age-dependent neurobehavioral deficits. Mitochondrial phenotype: Age-dependent increase in mtDNA deletions, reduced mtDNA copy number, mild bioenergetic defects. **Mitophagy phenotype:** reduced Parkin protein expression, increased LC3 protein expression. Twinkle^dup/+^; Parkin^−/−^ (TwinkPark): increased mtDNA deletions, reduced mitochondrial function (complex II activity) and compromised bioenergetics. Reduced membrane potential, neurobehavioral deficits, reduced striatal dopamine and increased TH-positive cell loss by 19 months.	(169, 170)
** Disruption of Key Mitochondrial Processes**				
ΔOTC	TH-Cre; ΔOTC Cre-mediated recombination in DA neurons	Exogenous expression of ΔOTC proposed to induce mitochondrial unfolded protein response (mtUPR) *in vivo* Ornithine transcarbamylase enzyme transgene (OTC normally restricted to liver) – Deletion mutant Δ30–114 Mitochondrially localized enzyme. Cre-recombination induces mitochondrial unfolded protein response in TH-positive neurons	**Neuronal phenotype:** mildly reduced motor function, reduced SNc dopamine content, decreased TH-positive neurons, L-DOPA responsive motor phenotype. **Mitophagy phenotype:** Pink1^−/−^*versus* Pink1^−/−^; ΔOTC: reduced DA neurons or reduction in DA content following ΔOTC expression [additive effects of PINK1 loss unknown—no comparison of ΔOTC alone *versus* Pink1^−/−^; ΔOTC], no L-DOPA responsive motor phenotype.	(171)
Ndufs4^−/−^	Mox2-cre: Ndufs4^LoxP^: Ubiquitous expression DAT-cre; Ndufs4^LoxP^: DA neuron expression	Conditional knockout of ETC complex I subunit, NDUFS4 Mitochondrial ETC complex I deficiency (activity and expression)	**Neuronal phenotype:** Ndufs4^−/−^: TH-positive cell loss, motor deficits, reduced striatal dopamine. Decreased 20S proteasome activity in SNc, decreased neurofilaments in SNc, increased ubiquitinated protein levels in DA neurons in SNc. DAT-cre; Ndufs4^loxP^: no motor deficits, slight decrease in TH-positive neurons at 24 months, no loss of DA nerve terminals, no overt neurodegeneration. Slight decrease in dopamine content and alterations to dopamine homeostasis in striatum. Reduced dopamine release. [Conflicting data around TH-positive cell loss, motor deficits, reduced striatal dopamine]. **Mitochondrial phenotype:** reduced complex I expression and activity. **Mitophagy phenotype:** small reduction in PINK1 expression, no change in Parkin expression	(237, 238, 239)

AAV, adeno-associated virus; CFP, cyan fluorescent protein; DA, dopaminergic; DAT, dopamine transporter; FRET, Förster resonance energy transfer; GFP, green fluorescent protein; mtDNA, mitochondrial DNA; MTS, mitochondrial targeting sequence; mtUPR, mitochondrial unfolded protein response; OTC, ornithine transcarbamylase; SNc, substantia nigra pars compacta; Tg, transgenic; TH, tyrosine hydroxylase; TOLLES, TOLerance of Lysosomal EnvironmentS; VTA, ventral tegmental area.

Despite limitations associated with each reporter (highlighted above), analysis of mitophagy dynamics *in vivo* has provided meaningful insight into mitochondrial quality control at an organismal level and holds significant potential for future study. Mitophagy reporters described to date cannot directly distinguish between PINK1–Parkin-dependent and -independent pathways. Full characterization and systematic comparison of loss-of-function (partial or full) or pathogenic mutations within key mitophagy regulators, including but not limited to *Pink1* and *Parkin*, are necessary in each model, especially given differences in reporter localization and behavior. Close examination of PD-related cells and tissues (DA neurons, astrocytes, gastrointestinal tissue), in combination with the correct “pathophysiological trigger” (see below), may be required to validate roles of mitophagy regulators *in vivo*.

### Disease modeling by genetically-induced mitochondrial dysfunction

Achievement of preclinical proof-of-concept for a potential therapy relies on the specific, mechanism-of-action (MoA)-governed efficacy in human biology-relevant disease models, such as transgenic, knock-in or knockout animals. Interestingly, there has been a striking failure to recapitulate neurodegeneration *in vivo* with *Pink1*^−/−^ and *Parkin*^−/−^, with a few exceptions (157, 158). Hypotheses as to these failures are discussed further below and include species differences, developmental adaptation due to germline ablation, or mitophagy-independent roles of these proteins. *Pink1*^−/−^ and *Parkin*^−/−^ knockout models themselves are not highly relevant in the search for novel enhancers of mitophagy. PINK1 and Parkin proteins are key targets and their complete loss of protein is not representative of most cases of human disease, though they will be of use to demonstrate target engagement or MoA. Instead, *in vivo* models with primary mitochondrial dysfunction may be of use. Genetic perturbation within key mitochondrial processes can serve as the pathological trigger to produce a neurodegenerative or aging phenotype and demonstrable alterations in mitophagy. These observations not only support a key role for mitochondrial dysfunction in aging and PD etiology but provide a model in which future therapeutics could be tested. However, given the complexity of PD pathophysiology, it may be unrealistic to expect one *in vivo* model to accurately recapitulate all elements of PD. Instead, recognition of an appropriate pathophysiological trigger may allow direct analysis of one or more factors contributing to the PD syndrome, whereby pharmacodynamic endpoints for compound-driven disease modification *via* mitophagy enhancement could be derived (Table 2).

### Disruption of mtDNA homeostasis triggers aging and neurodegenerative phenotypes *in vivo*

Disturbed mtDNA homeostasis is frequently observed in sporadic PD. By genetically perturbing key cellular regulators of mtDNA maintenance and transcription, either aging (a highly significant PD risk factor) or neurodegenerative phenotypes (characteristic of PD pathology) can be accurately recapitulated *in vivo*, depending on the mutation (Table 2). The Mutator mouse, a model in which a proof-reading deficient mitochondrial DNA polymerase γ (POLγA^D257A^) has been knocked-in, spontaneously accumulates mtDNA mutations (159). Although these mice exhibit no neurodegeneration and the nigrostriatal pathway remains intact up to 1 year (160), they display a dramatic aging phenotype. Aging is a significant risk factor for PD, and age-related decline in respiratory function and accumulation of mtDNA mutations, often observed in PD pathophysiology, is paralleled in the Mutator mice (159) (Table 2). Numerous studies have addressed mitophagy and the role of Parkin in this model (Table 2). Interestingly, neurodegeneration becomes evident by 12 months in POLγA^D257A^ (Mutator); Parkin^−/−^ mice, with evidence of midbrain DA cell loss and disrupted DA signaling and metabolism (161). Here, the appearance of neurodegeneration coincides with mitochondrial dysfunction, suggesting both a role of mitochondria in the degenerative phenotype and of Parkin in neuroprotection (161). No difference in mutational frequency is identified following Parkin knockout; however, a small but significant effect on mutation pathogenicity (*i.e.*, biological impact of mutations) was observed, suggesting Parkin selectively limits mitochondrial mutagenic stress. In addition, POLγA^D257A^ (Mutator); Parkin^−/−^ mice display a strong STING-dependent type 1 interferon inflammatory response, as measured by serum cytokines. This STING-dependent response is a cellular innate immune response to cytosolic DNA in hematopoietic and epithelial cells, hypothesized to be triggered through released mtDNA and sensed by cyclic guanosine monophosphate (cGMP)–adenosine monophosphate (AMP) synthase (cGAS) (156). In a cardiac model, restoring Parkin-mediated mitophagy in the Mutator hearts does not rescue the cardiac hypertrophy that develops with age in these mice, suggesting Parkin plays a minimal role in mtDNA mutation-induced cardiac aging (162).

A second *in vivo* model, the mitoPARK mouse, recapitulates cardinal clinical features of PD (163) (Table 2). Homozygous deletion of the mitochondrial transcription factor A (*Tfam*) gene within DA neurons causes mtDNA depletion and ETC deficiency, accompanied by classical PD features including age-related L-DOPA responsive motor dysfunction, mild cognitive impairment, intraneural inclusions, and rapidly progressive severe DA degeneration (163, 164, 165). The role of Parkin-mediated mitophagy has also been addressed in this model. Colocalization of Parkin with mitochondria was not observed in control or MitoPARK DA neurons following introduction of an AAV-encoded Parkin-mCherry vector directly to the midbrain. Additionally, loss of Parkin failed to affect the degree of mitochondrial aggregation or the progression of neurodegeneration, suggesting either Parkin cannot protect in a model in which mitochondria cannot replicate their genome or loss of mitochondrial membrane potential under these conditions is insufficient to recruit Parkin (161, 166).

PD-mito-PstI mice also exhibit mtDNA dysfunction-induced neurodegeneration (Table 2). A mammalianized gene for the *Pst*I restriction enzyme, with a human COX8A N-terminal MTS, is expressed under control of a dopamine transporter (DAT) promoter-driven tetracycline transactivator protein to allow spatiotemporal control. Expression of Mito-PstI causes double strand breaks by cutting the two *Pst*I restriction sites within mtDNA, producing large mtDNA deletions and mitochondrial dysfunction in DA neurons. Mice display a mild, slowly progressing parkinsonian phenotype, with an L-DOPA-reversible motor deficit and progressive degeneration of the DA neuron population within the SNc (167). Here, both motor and degenerative phenotypes are accelerated but not worsened by Parkin knockout (168).

The mitochondrial helicase Twinkle unwinds mtDNA during replication. A transgenic model creating in-frame duplication of amino acids 353 to 365 in Twinkle, under the TH promoter, causes mtDNA deletions in DA neurons, age-dependent progressive loss of DA neurons in SNc, and motor defects (169) (Table 2). Twinkle-duplication mice have slightly reduced Parkin expression, and indeed Parkin knockout (TwinkPark mice) increased mtDNA deletions and neurobehavioral deficits, reduced mitochondrial function, and accelerated nigral neurodegeneration (169, 170).

Neurodegeneration with mitochondrial origin has also been induced *in vivo* using strategies distinct from disruption of mtDNA homeostasis. For example, exogenous expression of a misfolding mutant of ornithine transcarbamylase (ΔOTC) in TH-positive neurons induces the mitochondrial unfolded protein stress response (171) and deletion of ETC complex I component, *Ndufs4* leads to ETC complex I deficiency (Table 2). Few genetic models have been effectively combined with mitophagy reporters, and data is conflicting regarding the significance of PINK1–Parkin-mediated mitophagy in these paradigms.

### Identifying the correct pathophysiological trigger to induce mitophagy

In the previous sections, direct mitochondrial dysfunction as a trigger for modeling PD has been discussed but inconsistencies in PINK1 and Parkin perturbation suggest that PINK1–Parkin physiological function *in vivo* is strongly context-dependent, and therefore, it may be necessary to explore this area more broadly.

Mitochondrial self-antigens can be presented at the cell surface following stress, a process known as mitochondrial antigen presentation (MitAP), to trigger an immune response. MiTAP relies upon derivation of mitochondrial vesicles, the formation of which PINK1 and Parkin have been proposed to repress (172). In *Pink1* knockout mice, intestinal infection with Gram-negative bacteria precipitates the promotion of mitochondrial antigens. Autoimmune mechanisms are triggered resulting in a neurological phenotype of both reduced striatal TH-positive axonal boutons in DA neuron axon terminals and an L-DOPA responsive motor deficit (173). These data underline the significance of PINK1 in autoimmunity in PD pathophysiology. In another interesting model, following administration of preformed fibrils in a α-synuclein seed injection model (an experimental model of α-synuclein cell-to-cell transmission and pathology), *Pink1* knockout rats demonstrate elevated levels of phosphorylated α-synuclein and greater vulnerability to nigral cell loss (174). Finally, a regimen of exhaustive exercise in mice induces a similar inflammatory response following *Parkin* knockout as does accumulation of mtDNA mutations in the Mutator mouse (156).

Together, these observations suggest that careful consideration is necessary to select the correct *in vivo* model for each therapeutic strategy, with the appropriate mitophagy-related deficit and pathophysiological trigger. Consideration must be given not only to how the pathophysiological trigger used relates to PD pathology and how representative the model is of disease, but also how that specifically relates to clinical presentation of patients with “mitochondrial” PD and to therapeutic mechanism of action. Knockout of either *Pink1* or *Parkin* will not provide a useful model if one aims to activate the pathway. Heterozygosity in either *Pink1* or *Parkin*, whether involving a disease-causing mutation or single allelic loss, may provide a partial loss-of-function haploinsufficiency model where enough residual activity remains for therapeutic rescue. Much like the Parkin-activating mutations described above, introduction of activating mutations into a model with a disease-relevant “rescuable” PINK1 or Parkin mutation would provide *in vivo* proof of concept evidence of disease modification. Although no published characterization is available to date, the Michael J Fox Foundation has sponsored the creation of a CRISPR/Cas9 knock-in mouse carrying the *Park2* W402A point mutation (W403A in human) in exon 11, which is available from the Jackson Laboratory (Parkin W402A KI; C57BL/6N-Prkn^em1Mjff^/J). Finally, small molecules (*e.g.*, USP30 inhibitors or PINK1 activators) may also be used to provide evidence of therapeutic benefit of mitophagy activation for preclinical proof-of-concept, assuming the correct model is identified.

Lack of significant phenotype in *Pink1* or *Parkin* knockout mice makes the functional contribution of PINK1 and Parkin to disease etiology challenging to understand. It remains possible that developmental compensation following germline ablation of key mitophagy genes and the dependence of mitophagic signaling on cellular context complicates conclusions. Furthermore, beyond mitophagy the full function of PINK1 and Parkin remains to be completely defined, with roles in neuroinflammation, mitochondrial biogenesis, and translation having been proposed (156, 175, 176, 177). Finally, given difficulties in translation between animal models and humans, including the temporal aspects of disease onset and progression in humans compared with aging of mice, particularly with respect to neurodegeneration, it may be appropriate to use human samples and relevant human disease cell models in parallel, such as patient-derived iPSCs. In either respect, strong evidence-based biomarkers are required to establish both preclinical and clinical proof-of-concept.

## Biomarkers for developing mitophagy-enhancing therapies in PD

Development of effective therapies for PD requires biomarkers to complement clinical assessment of patients. These biomarkers need to be clinically relevant, sensitive to therapeutic effects or underlying biology, and accessible to investigation. However, very few validated biomarkers of any kind are currently available for PD. For therapeutic trials in PD, biomarkers are needed at several stages, commencing with patient selection or stratification. Biomarkers of mitochondrial or mitophagic dysfunction are needed to identify those patients who are most likely to benefit from mitophagy enhancement therapies, improving efficiency and effectiveness of the trial. During a trial, biomarkers are needed to directly analyze target engagement, if possible, and also to measure downstream biological effects of potential therapeutics, to complement use of endpoints based on clinical measures. Finally, if specific side effects are expected from modulating mitophagy, biomarkers for these negative events are also important. Availability of biomarkers is an area of unmet need in the PD research field and much work is being done to identify and validate appropriate biomarkers, as reviewed elsewhere (178, 179). This section specifically discusses biomarkers that may be useful in developing therapies targeted at improving mitophagy and mitochondrial function in PD (Table 3).

**Table 3:** Potential biomarkers for mitochondrial dysfunction in PD

Biomarker	Description	Method	Potential use	References
**Mitophagy Proteins**				
phospho-PINK1 and phospho-Parkin	PD-associated mitophagy regulator proteins, which are phosphorylated during activation. Parkin is detectable in human serum.	Serum—ELISA	Stratification, efficacy	(69, 190)
phospho-Ser65-ubiquitin	Increased in sporadic PD postmortem brain and reduced in PINK1 and Parkin PD cases.	Plasma—ELISA	Stratification, efficacy	(187, 189)
Miro1	Miro1 clearance and degradation is disrupted in PD patient fibroblasts (including PINK1, Parkin, LRRK2 and sporadic patients).	Cell-based assay	Stratification	(27, 128)
**Oxidative Stress**				
Coenzyme Q10	The proportion of oxidizd coenzyme Q10 is elevated in PD plasma and platelets. PD patient lymphocytes have coenzyme Q10 deficiencies.	Blood—HPLC Lymphocytes—functional assays	Stratification, efficacy	(192, 193, 197, 240)
Oxidized DJ-1	Elevated in erythrocytes from unmedicated PD patients, whereas patients on dopamine replacement therapies had levels similar to controls. Increased in urine in PD compared with control.	Blood, urine—ELISA (using specific antibodies to oxidized form)	Stratification, efficacy (may require treatment-naive patients)	(194, 195)
8-OHdG	Elevated in CSF, serum and urine of PD patients compared with controls. Correlates with measures of disease severity.	CSF, serum, urine—HPLC, ELISA	Stratification, efficacy	(196, 197, 198, 200)
**mtDNA**				
mtDNA copy number	Reduced in SNc of PD brain and in blood cells of PD patients, though inconsistent between studies.	Blood (cell fractions)—qPCR	Stratification, efficacy	(201, 202, 203)
ccf-mtDNA	Reduced in PD CSF but increased in serum of PINK1 and Parkin PD patients.	CSF—qPCR	Stratification, efficacy (may require treatment-naive patients)	(204, 241, 242)
**Inflammation**				
IL-6	Elevated in serum of sporadic PD patients, *Parkin* PD patients, and *Parkin* heterozygotes. Some studies have found correlations between IL-6 levels and PD severity.	Serum—ELISA, cytokine immunoassays	Stratification, efficacy	(156, 204, 206, 207, 208)
**Imaging**				
Magnetic resonance spectroscopy (MRS)	Hydrogen nucleus (^1^H) MRS: N-acetylaspartate is reduced in PD patient brain and can be improved by dopamine replacement therapies. Lactate is elevated in PD patient brain. Phosphorus (^31^P) MRS: ratios of inorganic phosphate and ADP to ATP are increased in PD brain.	Magnetic resonance spectroscopy	Stratification, efficacy	(209, 210, 211, 219, 220)
FDG	A consistent pattern of altered brain metabolism is present in PD patients. FDG-PET changes are associated with disease progression and severity and can be used to analyze response to treatment.	PET	Efficacy	(214)
^18^F-BCPP-EF (mitochondrial complex I)	^18^F-BCPP-EF signal is reduced in monkeys treated with MPTP. In an initial study, no significant difference was seen in PD patients.	PET	Stratification, efficacy	(215, 216, 217, 218)
**Peripheral Function**				
Fibroblasts	PD patient fibroblasts have mitochondrial dysfunction and delayed mitophagy.	Cell-based assay	Stratification	(128, 224, 225, 226)
PBMCs	PBMCs of sporadic PD patients have elevated glycolysis, increased ROS production, and an increased proportion of damaged mitochondria.	Cell-based assay	Stratification, efficacy	(227, 228)

8-OHdG, 8-hydroxydeoxyguanosine; ccf-mtDNA, circulating cell-free mitochondrial DNA; CSF, cerebrospinal fluid; ELISA, enzyme-linked immunosorbent assay; FDG, fluorodeoxyglucose; HPLC, high-performance liquid chromatography; IL-6, interleukin 6; MPTP, 1-methyl-4-phenyl-1,2,3,6-tetrahydropyridine; MRS, magnetic resonance spectroscopy; mtDNA, mitochondrial DNA; PBMC, peripheral blood mononuclear cells; PD, Parkinson’s disease; PET, positron emission tomography; qPCR, quantitative polymerase chain reaction.

PD is a complex disorder with heterogeneity in pathophysiology and clinical presentation. This is clearest in monogenic PD patients; for example, Lewy bodies, a hallmark of PD pathology, are not seen in all PINK1, Parkin, and LRRK2-associated PD patients (180, 181, 182). Mitochondrial or mitophagy defects may be involved to a different degree in different patients and at different times. Stratification of patients to identify those with mitochondrial impairment, using clinical features, genetic risk, or biochemical markers, would allow selection of patients most likely to respond to mitophagy-enhancing therapies. Studies comparing sporadic PD to PINK1 and Parkin mutation carriers have been performed, but clinical presentation cannot distinguish between sporadic PD patients and those with specific genetic defects (183). Genetic risk could be used to identify patients who may have mitochondrial dysfunction, whether based on variation in known familial PD genes linked to mitochondria (particularly *PINK1* and *Parkin*) or on mitochondria-associated genes drawn from large-scale genetic studies of PD (184, 185). A panel of genes and variants with links to mitochondrial function could identify patients with higher likelihood of having defects in mitophagy or mitochondria and therefore with higher likelihood of responding to mitophagy-targeted therapies. This approach was taken in the design of a recent trial of coenzyme Q10 supplementation in PD, where patients are grouped using a “mitochondrial risk burden” based on PINK1, Parkin, and a panel of single-nucleotide polymorphisms (SNPs) identified from genome-wide association studies (186).

**Figure 3:**
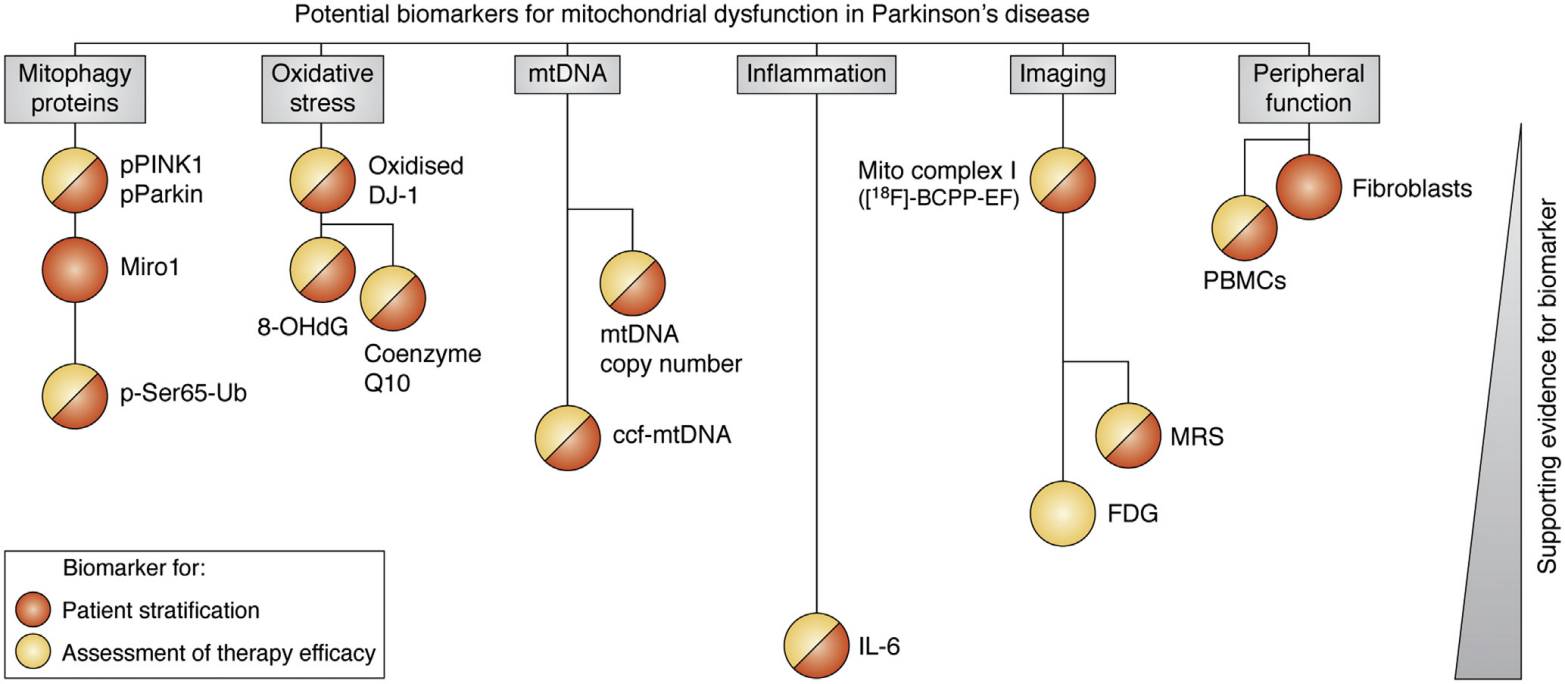
**Potential biomarkers for mitochondrial dysfunction in PD fall into several categories based on biological function.** Biomarkers may be useful for patient stratification (*red*), measuring therapeutic efficacy (*yellow*) or potentially both. Biomarkers are organized based on increasing levels of supporting evidence.

For therapies targeting PINK1–Parkin and other mitophagic pathways, the ideal biomarker would report directly on mitophagic activity and key protein targets (Fig. 3). Levels of mitophagic proteins, or the presence of PTMs including phosphorylation or ubiquitination, could provide such a marker. Phospho-Ser65-ubiquitin (Table 3), the result of PINK1-Parkin pathway activity, is elevated in postmortem brain tissue from sporadic PD patients, though in fixed tissue it is difficult to know whether this accumulation is the result of increased mitochondrial damage or reduced clearance (187). To date, PINK1 is the only known ubiquitin kinase (188), and therefore phospho-Ser65-ubiquitin could provide a specific biomarker of PINK1 activity and an indication of mitophagic flux. Watzlawik *et al.* (189) have developed a sensitive ELISA (enzyme-linked immunosorbent assay) for phospho-Ser65-ubiquitin, which can detect reduced phospho-Ser65-ubiquitin in PINK1 or Parkin knockout cell and mouse models, mutant PINK1 or Parkin patient fibroblasts, and in blood plasma from a very small number of patients carrying *PINK1* mutation. Another potential biomarker of the PINK1–Parkin pathway is Parkin itself, which is detectable in serum and CSF in other neurodegenerative diseases (190, 191). Parkin is phosphorylated as part of an activation sequence by PINK1 at serine 65 (69, 73, 74), an event that could act as a biomarker of PINK1–Parkin activity (Table 3). Ubiquitination and deubiquitination of mitochondrial proteins are key events in mitophagy; ubiquitination of TOM20, an OMM protein, has been proposed as a biomarker for USP30 inhibition (125), though it remains to be seen whether these events are detectable in accessible samples or only in cells. Another interesting example is Miro1 (Table 3), described above as a therapeutic target, which could act as a potential stratification marker for a specific therapy. The authors identified delayed removal of Miro1 from the OMM during mitophagy using PD patient fibroblasts, and they suggest that evaluation of Miro1 removal in fibroblast biopsies could be used to select patients who would benefit from treatment with a Miro1 reducer (27, 128). These proteins are promising candidates as proximal mitophagy biomarkers but are yet to be validated in patients.

Activating mitophagy should remove defective mitochondria and reduce oxidative stress, so oxidative damage may provide a surrogate measure of mitochondrial dysfunction and mitophagy enhancement (Fig. 3). The oxidation state of coenzyme Q10 is altered in PD patient platelets and plasma (192, 193) and oxidized DJ-1 (described above) is increased in erythrocytes (194) and urine (195) of PD patients (Table 3). Interestingly, in PD patients undergoing dopamine replacement therapy, the proportion of oxidized DJ-1 was similar to that in healthy controls (194). Oxidative DNA damage is also elevated in PD (Table 3). 8-hydroxydeoxyguanosine (8-OHdG), produced by oxidative damage of guanine bases in both nuclear and mtDNA, is elevated in CSF (196, 197, 198), serum (198), and urine (199, 200) of PD patients compared with healthy controls, and levels of 8-OHdG correlate with measures of disease severity (Table 3) (197, 199, 200). These biomarkers are pathophysiologically relevant and measurable in accessible patient samples.

mtDNA may provide another measure of mitochondrial dysfunction (Fig. 3). mtDNA copy number (Table 3), the number of copies of the mitochondrial genome per copy of the nuclear genome, is reduced specifically in SNc of PD post-mortem brain (201). While brain tissue is not suitable for biomarker development, reduced mtDNA copy number is also measurable in blood of PD patients, though results are not consistent (201, 202, 203). Circulating cell-free mtDNA (ccf-mtDNA), fragments of mtDNA released from cells following stress, is decreased in CSF of PD patients in some studies (Table 3) (201, 202), whereas serum ccf-mtDNA is increased in patients carrying *PINK1* or *Parkin* mutations (204). Some of the observed decrease in CSF ccf-mtDNA is associated with dopamine replacement therapies (201, 202), an interesting indicator that levels of ccf-mtDNA may respond to underlying biology, supporting its usefulness as a biomarker. Recently, reduction in methylation in the D-loop of mtDNA in PD brains compared with controls has been found (205), another potential indicator of mitochondrial involvement, but one that may be difficult to detect in patient samples. Enhancing mitophagy is hypothesized to have measurable effects on these markers and would enable measurement of treatment effect.

Inflammation is a contributor to PD pathogenesis and aspects of inflammation are influenced by mitochondrial function or by signaling networks involving Parkin (156, 206) (Fig. 3). Several inflammatory markers are elevated in PD (206). PINK1–Parkin defects increase STING-mediated inflammation, including production of interleukin 6 (IL-6) (156). IL-6 is increased in PD patient blood (206, 207, 208, 209, 210, 211), and studies have found correlations between IL-6 levels and PD severity (Table 3) (207, 208). Recent evidence further supports a specific link between IL-6 levels and PINK1–Parkin defects in PD. Borsche *et al.* (204) identified elevated IL-6 in PINK1 and Parkin patient blood compared with unaffected controls, whereas heterozygous carriers and sporadic PD patients only had a trend toward increased IL-6. They also found that IL-6 levels correlated with disease duration in PINK1 and Parkin patients, but not in sporadic PD patients (204). Parkin knockout also increases activation of the NLRP3 inflammasome in microglia and macrophages, leading to increased production of IL-1β and IL-18 in response to lipopolysaccharide in macrophages from *Parkin*^−/−^ mice and from a small cohort of PD patients with Parkin mutation (212). These inflammatory markers, particularly IL-6, may respond to altered Parkin function and are an interesting connection to other aspects of PD pathogenesis.

Imaging methods are a noninvasive way to obtain structural and functional data in the brains of living patients, a powerful tool for neurodegenerative diseases (Fig. 3). Single-photon emission computed tomography (SPECT) imaging of DA neurons with DaTSCAN, a radiolabeled ligand for the dopamine transporter, is approved in both Europe and the United States for use in PD diagnosis, though its usefulness as an endpoint in clinical trials is less clear (213). Imaging methods can also be used to monitor brain metabolism and potentially mitochondrial function. Fluorodeoxyglucose (FDG), a radiolabeled glucose analogue, is commonly used for positron emission tomography (PET) studies of metabolism or brain activity. PD patients have a distinct, complex pattern of changes in FDG-PET, which has been used extensively as a research tool in PD and may be useful in clinical trials (Table 3) (214). However, altered FDG-PET results from complex effects of cell loss and altered brain activity rather than specifically from changes to metabolism and mitochondrial function. Recently ^18^F-BCPP-EF has been developed as a radiotracer for mitochondrial complex I (Table 3) (215, 216). Monkeys treated with the neurotoxin MPTP, which causes a parkinsonian syndrome, had reduced complex I activity, which correlated with reduced dopaminergic signaling (217). In a small pilot study in early-stage PD patients, no significant difference was seen in ^18^F-BCPP-EF signal compared with controls (218) but, nevertheless, this is potentially a very interesting imaging tool for mitochondrial dysfunction in PD. Metabolism can also be assessed *via* magnetic resonance spectroscopy (MRS), a nuclear magnetic resonance technique commonly using either hydrogen (^1^H) or phosphorus (^31^P) nuclei to measure levels of prominent metabolites *in vivo* (Table 3) (219). MRS studies in PD patients have identified reduced N-acetylaspartate (209, 220, 221, 222), indicating neuronal loss or reduced neuronal metabolism, and elevated lactate (219) and an increased ratio of inorganic phosphate and adenosine diphosphate (ADP) to ATP (211, 223), indicating a reduction in mitochondrial energy metabolism. N-acetylaspartate levels improve in response to dopamine replacement therapies (220). These imaging methods could be used to analyze brain metabolism in PD patients and measure changes in response to mitophagy-enhancing therapies; indeed, the trial of coenzyme Q10 supplementation mentioned above will use ^31^P MRS to measure ATP metabolism as a secondary endpoint (186).

Mitochondrial function in peripheral cells, where detailed functional assays can be performed, could be used as a proxy for analyzing mitochondrial involvement or the impact of therapies in neurons. Fibroblasts from sporadic PD patients have mitochondrial dysfunction (224, 225) and impaired mitophagy (Table 3) (128, 226). One of these studies identified a correlation between fibroblast mitochondrial dysfunction and clinical measures, using statistical modeling to identify subgroups within their patient population, a method open to adaptation to stratify and select patients for clinical trials (225). Collection of skin biopsies is invasive, so using fibroblasts may be feasible for patient selection but not repeatedly over the course of a clinical trial. Mitochondrial function is affected in peripheral blood mononuclear cells (PBMCs) of sporadic PD patients (Table 3) (227, 228); however, it is unclear whether PINK1–Parkin mitophagy can be measured in these cells. PINK1 knockout has been shown to alter bioenergetics in PBMCs from rat (229), but a recent study suggests that the PINK1–Parkin pathway is not active in human PBMCs, and PINK1 may in fact not be expressed in these cells (230). Whether the PINK1–Parkin pathway is active in human PBMCs or not will determine their usefulness for analyzing mitophagy. Blood could be collected from patients over the course of a trial, but it remains to be seen whether mitochondrial dysfunction—or therapeutic effect—in peripheral cells will correlate with neuronal function or clinical outcomes.

These biomarkers may be able to analyze mitochondrial function in PD and some have potential to report directly on mitophagy, including phospho-Ser65-ubiquitin. Many of these are not specific to PD and have not been successful as diagnostic markers, but they could be useful for patient selection and measuring therapeutic efficacy. Work is still required to validate and characterize these biomarkers and develop them for clinical use. In future, approaches such as metabolomics and micro-RNA profiling (231, 232) could provide further biomarkers with links to metabolic and mitochondrial function in PD, increasing understanding of disease pathology and our ability to measure and target specific components of pathogenesis.

## Conclusions

Exploration of the cellular mechanisms underpinning mitochondrial quality control in human cells has prompted development of potential therapeutics for neurological disease. Human genetic studies have directed research into modulators of PINK1–Parkin-mediated mitophagy as a disease-modifying therapy for PD. Recent insights are beginning to identify further biological pathways and targets within mitophagy, spanning a wide range of physiological functions both dependent on and independent of Parkin and amenable to targeting by small molecule compounds. Many proteins and processes still require independent corroboration to establish their validity as therapeutic targets. In many cases, determination of whether a target protein is pathophysiologically relevant and amenable to therapeutic perturbation will also require careful consideration of any mitophagy-independent function. Furthermore, target validation will require understanding of the specific mechanisms that direct the target protein toward involvement in mitophagy, particularly with respect to E3-ubiqutin ligases, proteases, and kinases. Numerous small-molecule tool compounds are now available to aid in dissection of the pathway and potentially inform future drug discovery efforts. Beyond identification of the most efficacious intervention point and a potent, selective molecule, the path to clinic of disease-modifying therapeutics accelerating mitophagy does have several challenges in the development of suitable models and biomarkers. However, despite these obstacles and open questions, modulation of one or more of the many intervention points discussed may hold clinical potential. Further understanding the intricacies of mitophagy is an ongoing challenge for the PD community, but the answers will improve our knowledge of mitophagy and aid PD therapeutic development.

## Conflict of interest

E. C., A. V., T. H., and T. B. are all employees of Eisai Ltd.
